# Advances in the Development of New Drugs and Treatment Targets for Brain Cancers

**DOI:** 10.3390/brainsci14060526

**Published:** 2024-05-22

**Authors:** Luis Exequiel Ibarra, Laura Natalia Milla Sanabria, Nuria Arias-Ramos

**Affiliations:** 1 Instituto de Biotecnología Ambiental y Salud (INBIAS), Universidad Nacional de Rio Cuarto (UNRC) y Consejo Nacional de Investigaciones Científicas y Técnicas (CONICET), Rio Cuarto X5800BIA, Argentina; lmilla@exa.unrc.edu.ar; 2 Departamento de Biología Molecular, Facultad de Ciencias Exactas, Fisicoquímicas y Naturales, Universidad Nacional de Rio Cuarto, Rio Cuarto X5800BIA, Argentina; 3 Instituto de Investigaciones Biomédicas Sols-Morreale, Consejo Superior de Investigaciones Científicas-Universidad Autónoma de Madrid (CSIC-UAM), 28029 Madrid, Spain; narias@iib.uam.es

Brain tumors are a significant concern for the global medical community, with over 300,000 cases reported annually worldwide [1]. While some tumors are benign, many can become malignant and invade healthy brain tissue. Advances in diagnosis and treatment, such as improved imaging, targeted therapies, and minimally invasive surgeries, have improved outcomes and quality of life for patients. Despite these advancements, brain and other central nervous system (CNS) tumors are the fifth most common type of cancer and the most common among children [2].

Various criteria such as the location, type, grade, invasiveness, and potential spread of a brain tumor can restrict the success of its treatment. The blood–brain barrier (BBB) can hinder the efficacy of some medications, and tumors can become resistant to treatments with prolonged exposure [3]. Neurological impairment resulting from therapies such as surgery and radiation therapy can also affect a patient’s quality of life [4]. The problems highlight the intricate nature of handling brain tumors and the necessity for continuous research to enhance results. Ongoing research is focused on comprehending the biology of brain tumors, discovering new treatment targets, and creating breakthrough medicines like immunotherapy and tailored drug delivery systems. These endeavors show potential for enhancing results and increasing survival rates in the future.

This Editorial refers to the Special Issue “Advances in the Development of New Drugs and Treatment Targets for Brain Cancers”. The Special Issue features original research articles and review articles that discuss new therapy tactics and target medications, emphasizing the significance of brain tumors at cellular and molecular levels.

Nineteen manuscripts were submitted for consideration for the Special Issue, and all of them were subject to a rigorous review process. In total, ten papers were finally accepted for publication and inclusion in this Special Issue (seven articles and three reviews). The contributions are listed below:

1. Aguilar-García, I.G.; Jiménez-Estrada, I.; Castañeda-Arellano, R.; Alpirez, J.; Mendizabal-Ruiz, G.; Dueñas-Jiménez, J.M.; Gutiérrez-Almeida, C.E.; Osuna-Carrasco, L.P.; Ramírez-Abundis, V.; Dueñas-Jiménez, S.H. Locomotion Outcome Improvement in Mice with Glioblastoma Multiforme after Treatment with Anastrozole. *Brain Sci.* **2023**, *13*, 496. https://doi.org/10.3390/brainsci13030496
2. Caverzán, M.D.; Beaugé, L.; Oliveda, P.M.; Cesca González, B.; Bühler, E.M.; Ibarra, L.E. Exploring Monocytes-Macrophages in Immune Microenvironment of Glioblastoma for the Design of Novel Therapeutic Strategies. *Brain Sci.* **2023**, *13*, 542. https://doi.org/10.3390/brainsci13040542
3. Hernández-Cerón, M.; Chavarria, V.; Ríos, C.; Pineda, B.; Palomares-Alonso, F.; Rojas-Tomé, I.S.; Jung-Cook, H. Melatonin in Combination with Albendazole or Albendazole Sulfoxide Produces a Synergistic Cytotoxicity against Malignant Glioma Cells through Autophagy and Apoptosis. *Brain Sci.* **2023**, *13*, 869. https://doi.org/10.3390/brainsci13060869
4. Tamas, C.; Tamas, F.; Kovecsi, A.; Serban, G.; Boeriu, C.; Balasa, A. The Role of Ketone Bodies in Treatment Individualization of Glioblastoma Patients. *Brain Sci.* **2023**, *13*, 1307. https://doi.org/10.3390/brainsci13091307
5. Hoshimaru, T.; Nonoguchi, N.; Kosaka, T.; Furuse, M.; Kawabata, S.; Yagi, R.; Kurisu, Y.; Kashiwagi, H.; Kameda, M.; Takami, T.; et al. Actin Alpha 2, Smooth Muscle (ACTA2) Is Involved in the Migratory Potential of Malignant Gliomas, and Its Increased Expression at Recurrence Is a Significant Adverse Prognostic Factor. *Brain Sci.* **2023**, *13*, 1477. https://doi.org/10.3390/brainsci13101477
6. Toader, C.; Eva, L.; Costea, D.; Corlatescu, A.D.; Covache-Busuioc, R.-A.; Bratu, B.-G.; Glavan, L.A.; Costin, H.P.; Popa, A.A.; Ciurea, A.V. Low-Grade Gliomas: Histological Subtypes, Molecular Mechanisms, and Treatment Strategies. *Brain Sci.* **2023**, *13*, 1700. https://doi.org/10.3390/brainsci13121700
7. Alves, A.; Silva, A.M.; Moreira, J.; Nunes, C.; Reis, S.; Pinto, M.; Cidade, H.; Rodrigues, F.; Ferreira, D.; Costa, P.C.; et al. Polymersomes for Sustained Delivery of a Chalcone Derivative Targeting Glioblastoma Cells. *Brain Sci.* **2024**, *14*, 82. https://doi.org/10.3390/brainsci14010082
8. Ravi Kiran, A.V.V.V.; Kumari, G.K.; Krishnamurthy, P.T.; Johnson, A.P.; Kenchegowda, M.; Osmani, R.A.M.; Abu Lila, A.S.; Moin, A.; Gangadharappa, H.V.; Rizvi, S.M.D. An Update on Emergent Nano-Therapeutic Strategies against Pediatric Brain Tumors. *Brain Sci.* **2024**, *14*, 185. https://doi.org/10.3390/brainsci14020185
9. Lima, I.S.; Soares, É.N.; Nonaka, C.K.V.; Souza, B.S.d.F.; dos Santos, B.L.; Costa, S.L. Flavonoid Rutin Presented Anti-Glioblastoma Activity Related to the Modulation of Onco miRNA-125b Expression and STAT3 Signaling and Impact on Microglia Inflammatory Profile. *Brain Sci.* **2024**, *14*, 90. https://doi.org/10.3390/brainsci14010090
10. Arias-Ramos, N.; Vieira, C.; Pérez-Carro, R.; López-Larrubia, P. Integrative Magnetic Resonance Imaging and Metabolomic Characterization of a Glioblastoma Rat Model. *Brain Sci.* **2024**, *14*, 409. https://doi.org/10.3390/brainsci14050409

Glioblastoma (GBM) is the most aggressive type of glioma in adult patients and has the highest occurrence among malignant tumors. Patients typically have a limited survival span with conventional treatments, largely due to factors such as incomplete surgical resection and glioma cell infiltration, which contribute to a poor prognosis. Contribution 5 delved into the exploration of actin family genes as potential biomarkers for assessing brain invasion and distant recurrence in gliomas. The study uncovered the significant role of ACTA2 as a migratory factor in malignant gliomas, correlating with recurrence. Understanding the migratory mechanisms in malignant gliomas holds paramount importance for the development of forthcoming therapeutic strategies, with ACTA2 emerging as a promising candidate for targeted therapeutic interventions.

Various endeavors are currently being made in preclinical models of GBM to discover new molecular or cellular targets for treating malignant glioma. Estrogen receptors have been found in GBM tumor cells, suggesting a potential application of hormone-based therapies. While not a typical treatment for GBM, various studies suggest it may have a role in combination therapy. Contribution 1 evaluated the functional significance of anastrozole treatment’s anticancer effect by altering ERα and GPR30 expression in GBM xenografts. As a result, there was an improvement in walking movement, perhaps due to a decrease in the size of the brain tumor in the right motor region.

Contribution 10 aimed to discover new MRI and metabolomic indicators of GBM and their effects on healthy tissue utilizing a C6 glioma rat model. The authors studied an advanced-stage GBM tumor model by using in vivo multiparametric MRI evaluations and ex vivo metabolomic HRMAS MRS studies, due to the challenges posed by GBM and the growing recognition of the importance of multiparametric MRI in understanding its pathophysiology.

Recent studies have emphasized the cellular metabolism reprogramming process, which plays a crucial role in establishing the cellular microenvironment for tumor development and the invasion of GBM cells in normal brain tissue. Contribution 4 examined the potential use of ketones (KBs) and the glucose–ketone index (GKI) in predicting tumor aggressiveness in patients with GBM in a prospective clinical investigation, emphasizing novel biomarkers like KBs or GKI that are easier to measure.

Considering the composition of the GBM tumor microenvironment (TME), recent efforts have been placed on understanding the microenvironment surrounding tumor cells and the interaction between these cellular and acellular components in different preformed tumor niches to design new treatment options. Contribution 2 offers a comprehensive review of the pivotal role played by a primary cellular immune component in GBM, namely monocytes/macrophages. It elucidates how, over the past decade, this population has increasingly been recognized as a cell target in the formulation of novel therapeutic approaches. Within this field of study, Contribution 9 described a new intervention involving rutin on the viability and regulation of miRNA-125b and STAT3 expression in GBM cells. It also examined the impact on the inflammatory profile and STAT3 expression in microglia during indirect interactions with GBM cells. Its findings confirm the anti-glioma properties of the flavonoid, which can also influence microglia to adopt a more effective anti-tumor behavior, making it a potential candidate for supplemental treatment for GBM.

Drug repositioning is a successful strategy used to explore existing drugs for new clinical uses. Evidence suggests that it can enhance therapeutic effects by utilizing alternative cell death mechanisms like autophagy or ferroptosis, leading to improved anticancer effects and the activation of the immune system. Contribution 3 investigated the combined effects of melatonin with albendazole or albendazole sulfoxide on GBM cells to determine whether they have an additive or synergistic lethal effect. The authors discovered that the combination therapies resulted in a much higher rate of apoptotic and autophagic cell death in GBM. Albendazole and albendazole sulfoxide suppressed proliferation regardless of melatonin. The data support the further assessment of these various medication combinations as a viable method to assist in the treatment of GBM.

The scientific community has directed a considerable amount of attention towards both natural and synthetic chalcones, owing to their diverse range of reported biological activities, notably their demonstrated antitumor effects mediated through the inhibition of various molecular targets. In Contribution 7, novel nanoparticles (polymersomes) were developed as alternative drug delivery systems to facilitate the encapsulation and sustained release of these promising anti-GBM chalcone compounds, exhibiting notable selectivity against GBM cells.

This Special Issue also discusses various types of brain tumors in addition to GBM. Low-Grade Gliomas (LGGs) are a diverse group of brain tumors that develop from glial cells and are identified by their unique histopathological and molecular features. Contribution 6 thoroughly analyzes LGGs, detailing their subtypes, histological characteristics, and molecular components. By studying the World Health Organization’s grading system, 5th edition, more details were included due to a thorough understanding of new laboratory techniques, especially genetic analysis. Finally, Contribution 8 analyzed nanotechnology-based treatment options for childhood brain tumors in the revision. Pediatric brain tumors are the most common type of pediatric cancer and present a significant barrier for treatment due to their ability to spread to nearby tissues, limiting the effectiveness of surgery as the only treatment option. Nanotechnology delivery systems may efficiently penetrate the BBB. By including receptors that are highly expressed in both blood–brain barrier cells and cancer cells, these systems can differentiate cancer cells from healthy ones and target therapeutic drugs specifically to malignant cells.

This Special Issue requested manuscripts on novel therapeutic approaches and target medications as well as the significance of brain tumors at the cellular and molecular levels. We wanted to gather relevant expertise from experienced authors on the issue. Both the scholarly community and the general public can freely access the content upon publication.
